# Identification of Patients With Diabetes Who Benefit Most From a Health Coaching Program in Chronic Disease Management, Sydney, Australia, 2013

**DOI:** 10.5888/pcd14.160504

**Published:** 2017-03-02

**Authors:** Grace Delaney, Neroli Newlyn, Elline Pamplona, Samantha L. Hocking, Sarah J. Glastras, Rachel T. McGrath, Gregory R. Fulcher

**Affiliations:** 1 Department of Diabetes, Endocrinology & Metabolism, Royal North Shore Hospital, St Leonards, Sydney, NSW 2065, Australia; 2 University of Sydney, Northern Clinical School, Royal North Shore Hospital, St Leonards, NSW 2065, Australia; 3 Charles Perkins Centre, University of Sydney, Australia; 4 Kolling Institute of Medical Research, University of Sydney, Royal North Shore Hospital, St Leonards, NSW 2065, Australia.

## Abstract

**Introduction:**

Chronic disease management programs (CDMPs) that include health coaching can facilitate and coordinate diabetes management. The aim of this study was to assess changes in patients’ general knowledge of diabetes, self-reported health status, diabetes distress, body mass index (BMI), and glycemic control after enrollment in a face-to-face CDMP group health coaching session (with telephone follow-up) compared with participation in telephone-only health coaching, during a 12-month period.

**Methods:**

Patients with diabetes were enrolled in a health coaching program at Royal North Shore Hospital, Sydney, Australia, in 2013. Questionnaires were administered at baseline and at 3, 6, and 12 months, and the results were compared with baseline. Glycemic control, measured with glycated hemoglobin A_1c_ (HbA_1c_) and BMI, were measured at baseline and 12 months.

**Results:**

Overall, 238 patients attended a face-to-face CDMP session with telephone follow-up (n = 178) or participated in telephone-only health coaching (n = 60). We found no change in BMI in either group; however, HbA_1c_ levels in patients with baseline above the current recommended target (>7%) decreased significantly from 8.5% (standard deviation [SD], 1.0%) to 7.9% (SD, 1.0%) (*P* = .03). Patients with the lowest self-reported health status at baseline improved from 4.4 (SD, 0.5) to 3.7 (SD, 0.9) (*P* = .001). Diabetes knowledge improved in all patients (24.4 [SD, 2.4] to 25.2 [SD, 2.4]; *P* < .001), and diabetes distress decreased among those with the highest levels of distress at baseline (3.0 [SD, 0.4] vs 3.8 [SD, 0.6]; *P* = .003).

**Conclusion:**

Diabetes health coaching programs can improve glycemic control and reduce diabetes distress in patients with high levels of these at baseline.

## Introduction

Diabetes is a chronic disease associated with illness and premature death (1). As a consequence, poor quality of life and diabetes distress are frequently observed (2,3). Diabetes distress (defined as the psychological stress related to living with diabetes), a poor quality of life, and low levels of diabetes knowledge, may have a detrimental effect on diabetes self-management and glycemic control, which in turn can lead to an increased risk of complications (4,5). Although the effect of diabetes on patient quality of life and feelings of well-being is not routinely assessed in clinical practice, such information can assist in developing an individual approach to diabetes management. 

Education programs tailored toward diabetes self-management are effective ways to engage patients in management decisions and improve health outcomes (6,7). In one study, attendance at a diabetes group education session for 1 year improved knowledge of diabetes and problem-solving ability (8). Similarly, diabetes self-management programs were found to improve quality of life and diabetes distress in people with both type 1 and type 2 diabetes (9,10). Health coaching, an individualized educational approach to self-management through problem solving, and goal setting may be a useful adjunct to regular diabetes education to improve glycemic control and self-reported health status (11,12).

A chronic disease management program (CDMP) incorporating health coaching was introduced at Royal North Shore Hospital (RNSH) in Sydney in 2013. This approach differed from a traditional approach to group diabetes education because it was tailored to patient-set goals and targets. The aim of this study was to determine whether this education program led to a change in patients’ self-reported health status, diabetes distress, diabetes knowledge, body mass index (BMI), and glycemic control over a 12-month period of follow-up, regardless of whether health coaching was provided as a face-to-face session with additional telephone support or as telephone health coaching alone. We sought to identify the types of patients that might experience the greatest improvements in these measures and hypothesized that this information could lead to a more directed approach to enrollment in such programs.

## Methods

### Diabetes chronic disease management program

The health coaching program facilitated through the Department of Diabetes, Endocrinology and Metabolism at RNSH is funded through the Northern Sydney Local Health District CDMP. The CDMP is designed for people with specific chronic diseases (diabetes, coronary artery disease, chronic obstructive pulmonary disease, congestive heart failure, hypertension) who are at increased risk of hospital admission (13). The services provided through the program could include access to, and coordination of, health care appointments and additional support to enable people to more effectively self-manage diabetes to potentially reduce complications, improve health, and prevent hospitalization (14). All patients enrolled in the CDMP at RNSH were asked if they would like to participate in the health coaching program; just over half (53%) of invited patients took part in the program in 2013. The eligibility criteria for participation in the health coaching program were that patients be English-speaking, aged 16 years or older, and have type 1 or type 2 diabetes.

### Diabetes health coaching

The diabetes health coaching program at RNSH consisted of a face-to-face group education session, entitled the “Empowerment Program,” and telephone calls from a health coach (diabetes nurse educator) for 12 months after the group session. Alternatively, patients unable to participate in the face-to-face session could participate in telephone coaching in which they received an initial telephone call with educational information and follow-up telephone calls from the health coach for 12 months. The frequency of telephone calls depended on patient preference and ranged from weekly to 3 times a month. For most patients, telephone calls were made once per month.

The diabetes health coaching program used a diabetes conversation map to guide the initial face-to-face discussion or telephone call, which focused mainly on management of diabetes complications and risk-factors. Patients were provided with information and guidance on healthy eating, recommended levels of physical activity, and prevention of diabetes complications. In addition, information was provided on diabetes-specific health targets and recommendations for timing of visits to a primary care provider for review of progress toward targets and testing of blood glucose levels. Patients were encouraged to set goals related to management of their diabetes: for example, minutes of exercise per day, daily healthy eating aims, or medication adherence. The health coach provided guidance on how to develop strategies that would enable patients to achieve their goals. In subsequent telephone calls with the health coach, patients reported on their progress and evaluated their strategies for achieving their goals.

### CDMP health coaching patient assessments

To examine patients’ general knowledge of diabetes we used the Diabetes, Hypertension and Hyperlipidemia (DHL) knowledge instrument (15). In brief, this instrument was evaluated and validated in a population of patients with diabetes in Malaysia (15) and contains questions on patients’ understanding of the importance of glucose, blood pressure, and lipid control in minimizing the risk of diabetes complications. The DHL questionnaire consists of 28 questions, and each question that is answered correctly is given a score of 1; the maximum score is 28. Self-reported health status was assessed by using the first question of the Short-Form 36 Quality of Life Instrument (SF-1) (16,17); a score of 4 or higher (maximum score, 6) on the SF-1 indicates poor health-status (17). Diabetes distress was assessed by using the Diabetes Distress Scale (DDS) (18,19). An overall score (maximum score, 6) is determined by aggregating answers to 17 questions that each have a scale of 1 to 6; a score of 3 or higher on the DDS demonstrates moderate to severe diabetes distress (20). The DHL, SF-1, and DDS questionnaires were administered at 4 time points throughout the program — at baseline and at 3, 6, and 12 months. These questionnaires have been validated for use in the population of people with diabetes (16–20).

### Glycemic control, BMI, and medical history

All patients recruited into the Diabetes CDMP at RNSH had a baseline HbA_1c_ measurement (immediately before being referred for health coaching) and subsequently at their routine appointment for diabetes complications screening at 12 months. In addition, weight and height were measured to calculate BMI (calculated as weight in kilograms [kg] divided by height in meters [m] squared) at baseline and 12 months. Data were obtained from patient medical records to determine the presence or absence of dyslipidemia, hypertension or diabetes complications (retinopathy, neuropathy or chronic kidney disease (defined clinically by an estimated glomerular filtration rate [eGFR] <60ml/min/1.73m^2^). 

### Study design

An audit of longitudinal, prospectively collected data was carried out to investigate the impact of a diabetes health coaching program on patient outcomes during a 12-month period of follow-up. All patients participating in the health coaching program were living in the catchment area of RNSH, which is within the Northern Sydney Local Health District, one of Sydney’s areas of highest socioeconomic status (21). The study was approved by the local institutional review board, the Northern Sydney Local Health District Human Research Ethics Committee (reference no. RESP/16/58).

### Statistical analyses

The differences in patient-reported diabetes knowledge, health status, and diabetes distress over time were assessed by using Student’s paired *t* test for parametric data and Wilcoxon matched-pairs signed-rank test for nonparametric data. For repeated measures, 1-way ANOVA was used to analyze parametric data, and the Friedman test was used for nonparametric data. Differences between groups were analyzed using Student’s unpaired *t* test or the Mann–Whitney test, where appropriate. The change in HbA_1c_ and BMI was assessed by paired *t* test. Statistical analysis was carried out using GraphPad Prism Version 6 (GraphPad Software, Inc) and *P* < .05 was considered significant.

## Results

### Patient demographics and characteristics

From January through December 2013, 178 patients participated in the face-to-face health coaching session followed by telephone support, and 60 patients participated in telephone-only health coaching (Table). Most patients (97.1%) in the overall cohort had type 2 diabetes, and most of the cohort was older than 65 years. Approximately two-thirds of the participants in both groups were women.

**Table:** Characteristics of Patients Participating in Health Coaching, Diabetes Chronic Disease Management Program, Royal North Shore Hospital, Sydney, Australia, 2013

Characteristic^a^^,^ b	Face-to-Face Health Coaching^c^ (n = 178)	Telephone-Only Health Coaching^d^ (n = 60)	All Patients (n = 238)
Age, mean (SD), y	69.0 (9.6)	66.6 (10.1)	68.4 (9.8)
Female	119 (66.9)	38 (63.3)	157 (66.0)
Type 1 diabetes	6 (3.4)	1 (1.7)	7 (2.9)
Type 2 diabetes	172 (96.6)	59 (98.3)	231 (97.1)
Duration of diabetes, mean (SD), y	12.4 (6.9)	13.5 (7.1)	12.7 (6.9)
Takes insulin	40 (22.6)	28 (46.7)	68 (28.6)
Takes oral antihyperglycemic medication	149 (83.7)	50 (83.3)	199 (83.6)
HbA_1c_ at baseline, % (SD)	6.9 (1.0)	7.3 (1.2)	7.0 (1.1)
HbA_1c_ at 12 months, % (SD)	6.9 (1.0)	7.0 (1.1)	6.9 (1.0)
BMI at baseline, kg/m^2^ (SD)	29.3 (5.7)	31.1 (5.5)	29.8 (5.4)
BMI at 12 months, kg/m^2^ (SD)	29.2 (5.1)	30.1 (5.6)	29.5 (5.3)
**Comorbidities^e^**			
Hypertension	123 (69.1)	42 (70.0)	165 (69.3)
Dyslipidemia	163 (91.0)	54 (90.0)	217 (91.2)
**Diabetes complications**			
Retinopathy	20 (11.2)	10 (16.7)	30 (12.6)
Chronic kidney disease	57 (32.0)	20 (33.3)	77 (32.4)
Peripheral neuropathy	39 (21.9)	11 (18.3)	50 (21.0)

Abbreviation: BMI, body mass index; HbA_1c_, glycated hemoglobin A_1c_; SD, standard deviation.

a Data are number (percentage) unless otherwise indicated.

b Data obtained from patient medical records.

c Patients who received one in-person coaching session.

d Patients who received coaching by telephone.

e Data recorded at baseline from patient medical records.

The incidence of comorbidities was similar between groups, and the most frequent comorbid conditions were hypertension (69.3%) and dyslipidemia (91.2%). We found no difference in age or duration of diabetes between groups (Table). Conversely, patients in the telephone-only health coaching group had a higher HbA_1c_ (7.3%; *P* = .03) and greater BMI (31.1; *P* = .04) at baseline and were more likely to take insulin (46.7%; *P* = .001) (Table).

The proportion of patients who completed assessments at all time points was 31.1% (74 of 238). Patients who did not complete all assessments were significantly more likely to be younger (mean, 67.0 y; standard deviation [SD], 10.0 y) than those who completed all assessments (71.3 y [SD, 8.7 y]) (*P* = .003) and to have a higher BMI at baseline (30.2 [SD, 5.3]) than those who completed all assessments (28.8 [SD, 5.6]) (*P* = .02) but were similar otherwise. Patients who did not complete all assessments exhibited no difference in diabetes knowledge, self-reported health status, or diabetes distress at baseline compared to patients who completed all assessments. Evaluable data (ie, baseline and 12-month data) were available for 50.4% of patients (120/238). 

### General knowledge of diabetes

**Figure 1:**
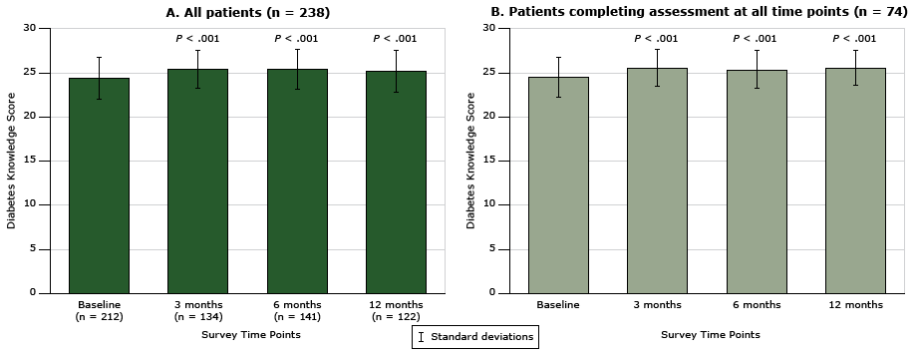
Change in patients’ general knowledge of diabetes over time, measured with the Diabetes, Hypertension and Hyperlipidemia (DHL) knowledge instrument (15), for patients participating in the health coaching program. The change in score (possible range, 0–28) was assessed over time in A) all patients (n = 238), and in B) patients who completed the assessment at all time points. Scores for A at each time point after baseline were compared with baseline scores by using the Wilcoxon matched-pairs signed-rank test. Scores for B at each time point after baseline were compared with baseline scores by using the Friedman test. Error bars indicate standard deviation. **Table:** 

The mean baseline score for general knowledge of diabetes for all participants who completed the baseline assessment (n = 212) was 24.4 (SD, 2.4). Diabetes knowledge improved significantly among participants who also completed the assessment at 12 months, from a mean score of 24.4 to a mean score of 25.2 (SD, 2.2) (*P* < .001) (Figure 1A). In addition, 74 patients (31.1%) completed the diabetes knowledge assessment at all time points; of these, 56 patients attended the face-to-face session, and 18 patients participated in the telephone-only coaching. General knowledge of diabetes improved over time whether or not participants completed all assessments (Figure 1A and Figure 1B). Diabetes knowledge improved in 3 months, and this improvement was sustained at one year post enrollment.

Time Point	Diabetes Knowledge Score, All Patients (N = 238)		Diabetes Knowledge Score, Patients Completing Assessment at All Time Points, Mean (Standard Deviation) (n = 74)
	No. of Respondents	Mean (Standard Deviation)	
Baseline	212	24.4 (2.4)	24.5 (2.3)
3 month	134	25.4 (2.1)	25.6 (2.1)
6 month	141	25.4 (2.3)	25.4 (2.1)
12 month	122	25.2 (2.4)	25.6 (2.0)

We found no difference in diabetes knowledge at baseline between patients who attended the face-to-face health coaching session and patients who participated in telephone-only coaching (mean score, 24.3 [SD, 2.5] vs 25.1 [SD, 1.8]; *P* = .10); however, patients who attended the face-to-face session improved significantly in diabetes knowledge at 12 months (mean score, 24.3 [SD, 2.5] vs 25.4 [SD, 2.4]; *P* < .001) compared with patients who participated in telephone-only coaching (mean score, 25.1 [SD, 1.8] vs 24.7 [SD, 2.4]); *P* = .66).

### Self-reported health status

**Figure 2:**
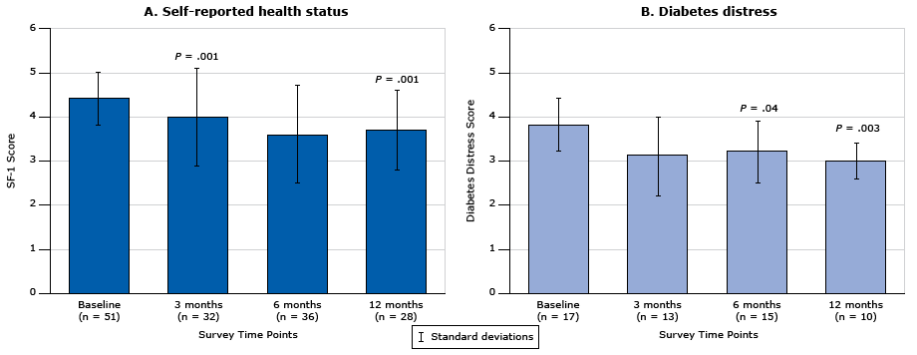
Change in general knowledge of diabetes over time in self-reported health status and levels of diabetes distress in patients with diabetes. A. Self-reported health status (measured by the Short-Form 36 Quality of Life Instrument [16,17]) among respondents who had a high baseline score (score of 4, 5, or 6) and who had completed a minimum of 2 assessments (n = 51, 21.4%). B. Diabetes distress among respondents who had moderate to high distress at baseline (Diabetes Distress Scale score >3) and who had completed a minimum of 2 assessments (n = 17, 7.1%); *P* = .04 for difference between 6 months and baseline; *P* = .003 for difference between 12 months and baseline. *P* values were calculated by using Wilcoxon matched-pairs signed rank test. Error bars indicate standard deviation. **Table:** 

The proportion of patients who reported poor health status at baseline (ie, patients with a score of ≥4 on the SF-1 scale) was 27.3%. Moreover, patients with diabetes complications (retinopathy, neuropathy, chronic kidney disease) were more likely to report a lower health status at baseline than patients with no diabetes complications (3.2 [SD, 1.0] vs 2.9 [SD, 1.0]; *P* = .01). Among patients with poor health status at baseline (n = 65) who completed a minimum of 2 assessments (n = 51), health status improved significantly for 36 patients from 4.4 [SD, 0.6] at baseline to 3.6 [SD, 1.1] at 6 months (*P* < .001) (Figure 2A) and for 28 patients from 4.4 [SD, 0.6] at baseline to 3.7 [SD, 0.9] at 12 months (*P* = .001) (Figure 2A). Patients taking insulin (n = 68) had the same health status score at baseline as patients taking an oral antihyperglycemic medication (3.2 [SD, 1.0] vs 3.0 [SD 1.0]). We found no difference in self-reported health status at any time point between patients who attended the face-to-face health coaching and patients who participated in telephone-only health coaching.

Time Point	Self-Reported Health Status (n = 51)		Diabetes Distress (n = 17)	
	No. of Respondents	Mean (Standard Deviation)	No. of Respondents	Mean (Standard Deviation)
Baseline	51	4.4 (0.6)	17	3.8 (0.6)
3 month	32	4.0 (1.1)	13	3.1 (0.9)
6 month	36	3.6 (1.1)	15	3.2 (0.7)
12 month	28	3.7 (0.9)	10	3.0 (0.4)

### Diabetes distress

Most patients who participated in the overall health coaching program (91.6%) had low levels of diabetes distress at baseline (DDS score <3). Patients with diabetes complications had distress levels similar to those without complications (DDS score, 1.9 [SD, 0.8] vs 1.9 [SD, 0.9]). Use of insulin was not associated with higher levels of distress (DDS score, 2.0 [SD, 0.9] vs 1.8 [SD, 0.8]; *P* = .13). Among patients who had moderate to high diabetes distress at baseline (DDS score >3) and who completed a minimum of 2 assessments (n = 17), we found a small yet significant decrease in diabetes distress at 6 months for 15 patients (DDS score, 3.2 [SD, 0.7] vs 3.8 [SD, 0.6]; *P* = .04) (Figure 2B) and 12 months for 10 patients (3.0 [SD, 0.4] vs 3.8 [SD, 0.6]; *P* = .003) (Figure 2B) compared with baseline. Similar levels of diabetes distress were found at each time point irrespective of health coaching method (face-to-face or telephone only).

### Glycemic control and body mass index

The average HbA_1c_ at baseline was 7.0% (SD, 1.1%), and 194 (81.5%) patients were considered to be at target HbA_1c_ level (<7%). For patients above target, that is, those with poor glycemic control at baseline (HbA_1c_ >7%; n = 44), we observed a significant improvement at 12 months (8.5% [SD, 1.0%] vs 7.9% [SD, 1.0%]; *P* = .03). The proportion of patients with poor glycemic control at baseline (HbA_1c_ >7%; n = 44) who achieved their target HbA_1c_ level at 12 months was 20.05%. We found no significant difference in the improvement in HbA_1c_ for patients based on health coaching method (Table).

We found no difference in BMI at 12 months compared with baseline in either the face-to-face or telephone-only health coaching group. Similarly, for patients with significant obesity at baseline (BMI ≥35; n = 29) we found no change in BMI at 12 months. Finally, health coaching method did not affect the change in BMI over time (Table).

## Discussion

The results of the present analysis identify patients who demonstrated the most benefit in diabetes distress and glycemic control after participation in a 12-month diabetes health coaching CDMP. Patients with a high level of diabetes distress, poor self-reported health status, and a low level of diabetes knowledge at baseline had the most improvement in these measures at both 6 and 12 months post CDMP initiation. The face-to-face health coaching session in the context of a CDMP resulted in significantly improved general knowledge of diabetes, whereas telephone health coaching only did not. Furthermore, patients with HbA_1c_ levels above target at baseline demonstrated a significant improvement in glycemic control.

Identification of patients who are likely to receive the most benefit from participating in a CDMP or health coaching is of clinical significance, because programs can be tailored toward this patient population, and screening can be initiated before their enrollment so that outcomes can be maximized and costs to the health care provider minimized. This identification will provide a direct benefit for patients and will also ensure that health care services are used efficiently. Moreover, additional measures could be put in place to target the areas in which patients report they are most distressed or require support and education for diabetes. Therefore, we focused our analysis on patients with low levels of self-reported health status, elevated diabetes distress, or HbA_1c_ levels above target at baseline. Health coaching is an innovative approach to chronic disease management that allows patients to set their own health goals, rather than the traditional approach of the provider setting the goals for the patient. This approach allows patients to feel more empowered and in control of their own health and may contribute to the improvement in psychological stress associated with diabetes, including perceived health-status and diabetes distress.

Our results are similar to those of Beverley et al, who found that older patients (aged 60–75 y) demonstrated positive changes in quality of life and diabetes distress after diabetes education in behavioral interventions (22). In addition, a recent study showed that health coaching for patients with type 2 diabetes resulted in improved satisfaction with life and a reduction in symptoms of depression (23). A meta-analysis determined that self-management and education interventions for diabetes were most effective in patients with depression symptoms or high levels of stress at baseline (24), which is similar to our findings. In contrast to other studies (7,25), we did not observe an improvement in glycemic control for the whole cohort after participation in the CDMP. The most likely explanation is that in our cohort, most participants (81.5%) had achieved target HbA_1c_ levels before commencing the program and maintained this level of glycemic control over the subsequent 12 months, despite an anticipated decline in β-cell function during follow-up. For patients with suboptimal glycemic control at baseline, a significant improvement was observed after health coaching, suggesting that this mode of diabetes education is of substantial benefit to patients with inadequately controlled blood glucose levels.

Diabetes knowledge and attitudes toward diabetes can directly affect self-management, which in turn influences quality of life (26). Consistent with this, we found improvements in both diabetes knowledge and diabetes distress, suggesting that greater understanding of diabetes may reduce the stress that accompanies it and may make dealing with diabetes easier for patients. Furthermore, patients provided positive feedback regarding the program and stated that a health-coaching approach to patient follow-up was beneficial.

Our study population had high levels of diabetes education at entry into the health coaching program; therefore, only a small proportion of patients had high levels of diabetes distress or poor glycemic control at baseline. As a result, the improvement that occurred was observed in a subset of the entire cohort of participants. However, the change in distress observed for this group was significant, at both 6 and 12 months after beginning health coaching, suggesting that a sustained improvement took place. Conversely, the change in self-reported health status over time may be due to response bias, because patients completed the same questionnaire at several time points throughout their participation in the health coaching program. Furthermore, because the frequency of follow-up telephone calls as part of health coaching was determined by patient preference, more regular contact with the health coach possibly resulted in improved outcomes. However we were unable to assess this in the present study.

The strengths of this study are the large number of participants and the longitudinal data capture as part of the health coaching program’s patient assessments. Our study had several limitations. A high attrition rate in questionnaire completion may have led to bias in the responses obtained. Furthermore, there was no control group in which diabetes health coaching did not take place, and the study was not randomized to health coaching compared with standard diabetes education. Nonetheless, each participant acted as his or her own control as the change in his or her questionnaire results, BMI, and glycemic control was measured over time and compared with baseline. There is also a potential limitation in the sampling method used, and the study participants may not be representative of the larger population of patients living with chronic conditions. However, we consecutively recruited agreeable patients into the health coaching program after their enrollment in the CDMP, and patient characteristics were representative of those in other cohorts of patients with diabetes in Australia (27,28).

In summary, patients with high levels of diabetes distress or poor glycemic control at entry into health coaching were the ones who benefitted most from taking part in the program. On the basis of our results, we propose that such patients be routinely offered additional health coaching or more specialized chronic disease management, rather than providing all patient populations with interventions that may not be relevant or efficacious. Personalized medicine is increasingly being recognized as an effective way of delivering health care, and identification of patients with diabetes most responsive to particular education strategies will contribute toward the optimization of health care resources.
